# Influence of Geomagnetic Disturbances at Different Times of Day on Locomotor Activity in Zebrafish (Danio Rerio)

**DOI:** 10.3390/clockssleep3040045

**Published:** 2021-11-29

**Authors:** Viacheslav V. Krylov

**Affiliations:** Papanin Institute for Biology of Inland Waters, Russian Academy of Sciences, 152742 Borok, Russia; kryloff@ibiw.ru; Tel./Fax: +7-485-472-4042

**Keywords:** geomagnetic activity, locomotor activity, circadian rhythm, zeitgeber

## Abstract

The influence of magnetic fields and natural geomagnetic storms on biological circadian rhythms are actively studied. This study reveals an impact of local natural perturbations in the geomagnetic field that occurred at different times of the day on circadian patterns of locomotor activity of zebrafish. A decrease in zebrafish swimming speed was observed during the geomagnetic disturbances before or after the fluctuations of diurnal geomagnetic variation. However, if the geomagnetic perturbations coincided with the fluctuations of diurnal geomagnetic variation, the decrease in zebrafish swimming speed was insignificant. This result suggests that the biological effects of geomagnetic disturbances may depend on synchronization with the diurnal geomagnetic variation. It implies that the previously published correlations between geomagnetic activity and medical or biological parameters could result from a disruption in circadian biorhythms.

## 1. Introduction

Zebrafish (*Danio rerio*) is a model organism widely used to study various biological processes. Locomotor activity of this species is a commonly used behavioral endpoint [1]. *D. rerio* inherently increased swimming activity during the day (light cycle) and have a sleep-like behavior during the night (dark cycle). Changes in the circadian pattern of zebrafish locomotor activity are used for neurophysiological studies and are suggested to be a tool for toxicity assessment [2,3,4,5,6]. In this regard, the evaluation of natural factors influencing the locomotor activity of zebrafish is of significant interest.

Researchers rarely take into account geomagnetic disturbances. However, there are reports on the influence of geomagnetic perturbations on the motor activity of animals. They affect the locomotor activity of rats [7] and king crab *Paralithodes camtschaticus* [8]. Significant changes in the magnetic background influence the swimming of Atlantic salmon *Salmo salar* juveniles [9] (Varanelli and Mccleave 1974). On the other hand, Deshcherevsky et al. [10] inform about the lack of correlation between the indices of geomagnetic activity and locomotor activity of catfish *Hoplosternum thoracatum* and cockroaches *Blaberus craniifer*. The issue of the influence of natural geomagnetic disturbances on the locomotor activity of zebrafish remains obscured.

One of the most probable hypotheses of the influence of geomagnetic disturbances on organisms implies that the diurnal geomagnetic variation [11] is a secondary zeitgeber for biological circadian rhythms [12,13,14]. This hypothesis was suggested in 1960 when it was found that biological circadian rhythms could be influenced by magnetic fields [15,16]. Currently, it is known that static and weak radiofrequency magnetic fields significantly slow down the cockroach circadian rhythm of locomotor activity under dim UV light [17]. The static magnetic field of 300 μT also slowed the rhythms of locomotor activity in *Drosophila.* However, there were also some flies with shortened periods [18]. A phase shifting and a significantly increased acrophase of locomotor activity rhythms were registered in rats exposed to radiofrequency magnetic fields [19]. Beyond the behavioral rhythms, magnetic fields altered circadian clock gene expression [20,21]. It was reported that exposure of zebrafish fibroblast cells to magnetic fields caused an increase in *Cry1aa* expression and the shifting of *Cry1aa* and *Per1b* oscillations phase, while those of *Clock1a* and *Per2* remained unaffected. Similarly, the exposure of larvae to the same magnetic fields between 11 and 14 days post fertilization led to the desynchronization of *Cry1aa* and *Per1b* circadian oscillations [22]. Moreover, preliminary data on the entraining of zebrafish circadian rhythms by magnetic oscillations with a period of 26.8 h are available [23].

An organism may perceive substantial geomagnetic perturbations as a disruption of diurnal geomagnetic variation. Biological effects arise from the inconsistency of processes synchronized with different zeitgebers: the primary light-dark cycle and the secondary diurnal geomagnetic variation disrupted by geomagnetic disturbances [24]. According to this hypothesis, the response of an organism to geomagnetic activity should depend on the synchronization between local perturbations in the geomagnetic field and the diurnal geomagnetic variation or, more simply, on the time of day when the disturbances occurred. However, there is a lack of studies testing this assumption.

This study analyzed 106-h video records of zebrafish movements from 31 July 2020 to 15 September 2020. Six local geomagnetic disturbances caused by perturbed solar wind occurred during the recording. It allowed comparing the effects of natural geomagnetic disturbances that occurred at different times of the day on the locomotor activity of zebrafish.

## 2. Results

A decrease in zebrafish swimming speed, increase in the percentage of freezing time, and decrease in the percentage of swimming time are observed as a general trend during the geomagnetic disturbances, with local K-index = 4 compared to the values obtained from the same fish during the magnetically quiet period (Table 1). The mean percentages of freezing and swimming time changed significantly under the disturbances that occurred before the morning fluctuations of diurnal geomagnetic variation on 31 August 2020 and disturbances after the morning fluctuations of diurnal geomagnetic variation on 2–3 August 2020 and 31 August 2020. The decrease in average swimming speed was statistically significant under disturbances that occurred before the morning fluctuations of diurnal geomagnetic variation on 31 August 2020 and disturbances after the morning fluctuations of diurnal geomagnetic variation on 2–3 August 2020. The same effect on the margin of significance was revealed under disturbances before the morning fluctuations of diurnal geomagnetic variation on 14 September 2020 (Table 1). However, there was no significant influence on swimming speed, percentage of freezing time, and percentage of swimming time if the perturbations in the geomagnetic field coincided with the morning fluctuations of diurnal geomagnetic variation (31 August 2020). Unsmoothed double plotted actograms of zebrafish swimming speed can be found in Supplementary Figure S1.

Studied disturbances did not significantly affect averaged meandering. There was a weak trend towards a decrease in the wall preference index during geomagnetic disturbances. On average, zebrafish significantly prefer visiting the central zone of aquaria under the disturbances that occurred before the morning fluctuations of diurnal geomagnetic variation on 31 August 2020 and disturbances after the morning fluctuations of diurnal geomagnetic variation on 2–3 August 2020.

It should be noted that the direction of changes in studied behavioral characteristics under disturbances that occurred before or after the morning fluctuations of diurnal geomagnetic variation was the same in all individuals for most cases (Table 1).

Table 2 contains summarized data for the events before or after the morning fluctuations of diurnal geomagnetic variation from different time intervals. The effects of events that occurred after the morning fluctuations of diurnal geomagnetic variation, in summary, were more expressed than those that occurred before.

## 3. Discussion

The decrease of locomotor activity in zebrafish during geomagnetic disturbances is consistent with earlier data in other species. It is known that various magnetic influences can lead to a reduction of locomotor activity in mice and rats [25,26,27], planarian [28], and fruit flies [29]. However, all these findings were obtained in artificial magnetic environments reproduced in a laboratory, while the present study results are based on natural geomagnetic disturbances.

The insignificant modulations of studied parameters by the local geomagnetic disturbances at the morning fluctuations of diurnal geomagnetic variation and notable effects at other hours can be evidence for the hypothesis that an organism may perceive substantial geomagnetic perturbations as a disruption of diurnal geomagnetic variation. Perhaps cryptochromes could be a link between magnetic influence and locomotor activity in zebrafish. On the one hand, cryptochromes are suggested to be responsible for the biological effects of geomagnetic storms [24] and magnetic-compass orientation in living beings [30]. On the other hand, cryptochromes are involved in the transcription-translation feedback loop as the main elements of the molecular circadian oscillator that manage the rhythms of locomotor activity [31,32]. A possible mechanism of magnetic bioeffects is based on changes of the singlet-triplet state of electrons in cryptochrome’s radical pairs, modulating the functional state of these proteins [33]. These magnetic-field-induced changes in the functional state of cryptochromes may, in turn, affect the repressor functions of the CRY:PER dimers and modulate the state of the transcription-translation feedback loop. However, it should be noted that only one case of coincidence of geomagnetic disturbances and fluctuations of diurnal geomagnetic variation was analyzed. Moreover, the geomagnetic disturbances on 31 August occurred by series, and geomagnetic activity was also recorded before and after fluctuations of diurnal geomagnetic variation on this day. More research is needed to clarify this issue.

More pronounced effects caused by the geomagnetic disturbances that occurred after the morning fluctuations of diurnal geomagnetic variation compared to that caused by the events that occurred before can be evidence for different efficiency of geomagnetic disturbances at different times of the day. At the same time, the differences in the activity of zebrafish at the light and dark phases should be taken into account. *D. rerio* show increased swimming activity during the light phase and sleep-like behavior during the dark phase [2,34]. The events before the morning fluctuations of diurnal geomagnetic variation occurred at the dark phase. It could be a reason for less pronounced effects than the events happening at the light phase.

## 4. Materials and Methods

All animal experiments were carried out following relevant guidelines and regulations. The Animal Care and Use Committee at the Papanin Institute for Biology of Inland Waters has approved the experimental protocols.

Wild-type zebrafish (AB strain) were obtained from the commercial distributer (Priroda, Yaroslavl, Russia) and maintained in the Laboratory of Physiology and Toxicology (Papanin Institute for Biology of Inland Waters, Russian Academy of Sciences). Before experimentation, zebrafish were kept together for two months in 70 L aquaria at 24 °C under a 16:8 h light/dark cycle. Zebrafish were fed daily at different times between 12:00 and 16:30. Males and females at the age of approximately four months (2.99 ± 0.17 cm, 0.26 ± 0.02 g, n = 24) were used for experimentation. Each zebrafish was used only for a single replication.

All experimentation was conducted in a remote laboratory free of working staff in order to eliminate possible circadian rhythm influences caused by daily human activities. Four fish were placed in four custom glass aquaria (15 × 20 cm, height: 23 cm) filled with 10 cm of water, with one fish per aquarium. Water temperature during the experiments was 21 °C, as adult zebrafish show the most robust rhythm of locomotor activity at the temperatures of 20–21 °C [35,36]. The aquaria were installed above a backlit lightbox. The lightbox was constructed from a series of LEDs, aluminum plates, and matte plexiglass. LED plates were created by adhering 32 LEDs to an aluminum plate so each aquarium would be backlit by 4 infrared LEDs (3 W, 940 nm) and 4 white-color LEDs (3 W, 4500 K). Each LED plate was mounted 10 cm under a lightbox cover from matte plexiglass that diffused light. Lighting modes were controlled via time relays (DH-48S-S, Omron, Japan), which used KMI-10910 (IEK, Russia) contactors to supply power, by Qh-60LP18 power suppliers (Shenzhen Chanzon Technology, Shenzhen, China), to the LEDs. Screens made of opaque white plastic were placed between the adjacent aquaria so that fish could not see conspecifics.

The water was constantly renewed via two 4 mm openings in the wall of each aquarium at a 3 and 10 cm height from the bottom. The water pH was 7.38, conductivity 500–680 mS/cm, the dissolved oxygen level was 7.1 mg/L, hardness 120 mg CaCO_3_/L, and alkalinity 80 mg CaCO_3_/L. Water flowed by gravity from a 200 L plastic barrel placed one floor above through the silicone hoses connected to the bottom openings of aquaria. Water aeration and temperature control for all aquaria were carried out in the barrel. Excess water was drained to the sewer through the top opening to ensure a constant level of 10 cm. Water from different aquaria has never been mixed or reused.

At the beginning of the experiment, a 1 cm^3^ piece of slow-release gel food block “Tetra Holiday” (Tetra GmbH, Melle, Germany) was placed on the bottom of each aquarium to prevent the influence of the feeding schedule on circadian behavior. Thereby zebrafish had free access to food during the whole study.

Fish movements in the horizontal plane were registered with IP-cameras (TR-D1140, Trassir, Shenzhen, China) equipped with IR corrected varifocal lenses (TR-L4M2.7D2.7-13.5IR, Trassir, Shenzhen, China) and mounted above the aquaria. Night and day video was recorded in black and white at 25 frames per second with a 2592 × 1520 pixels resolution. The video signals were transmitted through a switch (T1500-28PCT, TP-Link, Shenzhen, China) to a video recorder server (MiniNVR AF16, Trassir, Shenzhen, China).

**Figure 1:**
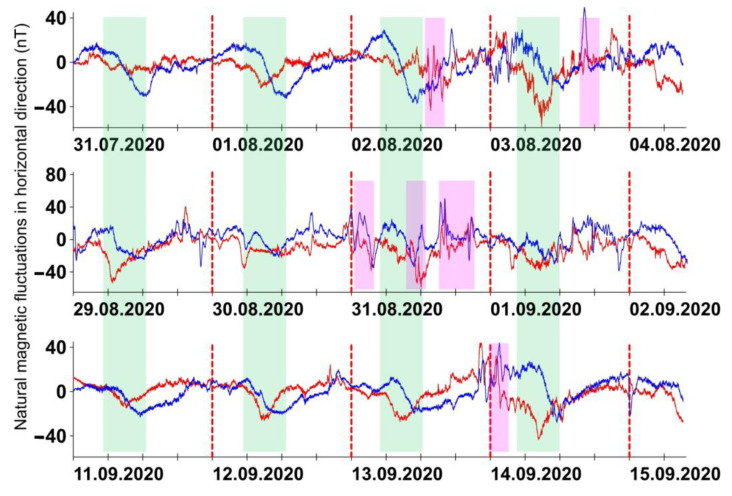
Dynamics of the geomagnetic field in the latitudinal (red line) and meridional (blue line) directions close to the site of experimentation. High green bars match fluctuations of diurnal geomagnetic variation. Low magenta bars match geomagnetic disturbances with K-index = 4.

Registration of the fish movements was carried out in the following time intervals: from 00:00 on 31 July 2020 to 10:00 on 4 August 2020; from 00:00 on 29 August 2020 to 10:00 on 2 September 2020; from 00:00 on 11 September 2020 to 10:00 on 15 September 2020 (Figure 1). The 106-h video records obtained from 12 zebrafish were then processed and analyzed.

The K index was used as a local characteristic of geomagnetic activity. It calculates for a specific geomagnetic observatory and varies in integer values from 0 to 9. The K index is derived from maximum fluctuations in the two horizontal geomagnetic components (H and D) after eliminating regular variation [37]. Six geomagnetic disturbances with a local K-index of 4 occurred at different times of the day during these time intervals (data from the Geophysical observatory “Borok”, located close to the site of experimentation and Pushkov Institute of Terrestrial Magnetism, Ionosphere and the Radio Wave Propagation Russian Academy of Sciences, located in Troitsk and about 300 km from the site of experimentation). These geomagnetic disturbances are described as very close in parameters. After eliminating regular variation, the range of fluctuations was 50–60 nT and differed insignificantly between six evets. In synchronization with the morning fluctuations of diurnal geomagnetic variation, these disturbances were divided into three: occurred before (31 August 2020 from 00:00 to 03:00 and 14 September 2020 from 00:00 to 03:00), after (2 August 2020 from 12:00 to 15:00, 3 August 2020 from 15:00 to 18:00, and 31 August 2020 from 15:00 to 21:00), and coincided (31 August 2020 from 09:00 to 12:00). The measurements carried out at the location of the experimental aquariums using an NV0302A magnetometer (ENT, St Petersburg, Russia) showed that LEDs, cameras, and other equipment did not generate any low-frequency magnetic fields in the range of 0–20 Hz that corresponds to the studied natural fluctuations.

An approach proposed by Audira et al. [2] was used for data processing. One-minute video files were cut from the primary video record for every half of an hour (from the 15th to the 16th and 45th to 46th min of each hour). The open-source software idTracker [38] was used to process each one-minute video file. The software provided X and Y coordinates reflecting the center of the fish body for each frame. Before the processing, the trajectory data were filtered using the “minimal distance moved” method to eliminate slight “apparent” movements of the fish [39,40]. The minimal distance threshold was set at 2.6 mm. Then, based on this information, the following quantitative measures of fish behavior were calculated using the Microsoft Excel formulae:

- Average swimming speed, cm/s (distance travelled divided by observation time);
- Meandering, °/cm, (sum of all turning angles divided by total distance);
- Freezing time, % (time when speed is less than 1 cm/s);
- Swimming time, % (time when speed is 1–10 cm/s);
- Rapid movement time, % (time when speed exceeds 10 cm/s);
- Wall preference index (relative time spent within a 3 cm-wide area close to the walls).

Initial data can be found in Supplementary Files. 

The analyzed parameters had a pronounced circadian rhythm. The use of correlation analysis for comparing magnetograms and locomotor activity was inappropriate, as circadian changes in analyzed parameters should correlate with the diurnal geomagnetic variation. For this reason, we used the following approach. The behavioral parameters for 3-h time intervals when K-index = 4 were compared with the parameters obtained from the same time interval on magnetically quiet days within each of the time intervals. Since the data were distributed normally, the differences between the means were assessed using a paired *t*-test. The comparisons were performed between the average values obtained from each individual within the hours when the geomagnetic disturbances occurred and the averaged values obtained from the same individuals within the same hours on quiet geomagnetic days.

## 5. Conclusions

The present results suggest that the biological effects of geomagnetic disturbances may depend on their synchronization with diurnal geomagnetic variation, i.e., on the time of day when geomagnetic activity occurs. Thereby, the insignificant influence of geomagnetic disturbances coincided with the morning peak of diurnal geomagnetic variation on organisms can interfere with meaningful effects in specific time windows when studying the biological impact of geomagnetic activity in time-series analyses. The present study results and other works [13,41] imply that the previously found correlations between geomagnetic activity and medical or biological parameters can be revised. This revising of long-term time series may consist of a separate estimation of the effects of geomagnetic disturbances that occurred during different time intervals during the day (e.g., morning events, afternoon events, evening events, and night events). However, one should consider the seasonal variability of the diurnal geomagnetic variation at different locations [42] to relate slow perturbations in the geomagnetic field with the morning fluctuations of diurnal geomagnetic variation.

## Figures and Tables

**Table 1:** Parameters of locomotor activity in zebrafish under geomagnetic disturbances (above dash) and at the same time on magnetically quiet days (below dash).

Individuals	Time Interval 1	Time Interval 2			Time Interval 3
	Events after FDGV 12:00–15:00 2 August 2020 15:00–18:00 3 August 2020 n = 12	Event before FDGV 00:00–03:00 31 August 2020	Event Coincided with FDGV 09:00–12:00 31 August 2020	Events after FDGV 15:00–21:00 31 August 2020	Event before FDGV 00:00–03:00 14 September 2020
Average swimming speed (cm/s)					
Fish#1	2.63 ± 0.19 3.44 ± 0.12	0.35 ± 0.09 2.28 ± 0.30	2.87 ± 0.56 2.49 ± 0.13	2.06 ± 0.15 2.05 ± 0.14	0.75 ± 0.17 0.76 ± 0.09
Fish#2	1.55 ± 0.09 1.65 ± 0.08	0.68 ± 0.15 1.14 ± 0.12	2.19 ± 0.18 2.66 ± 0.16	2.00 ± 0.40 1.86 ± 0.14	0.39 ± 0.09 0.67 ± 0.06
Fish#3	1.78 ± 0.07 2.05 ± 0.06	0.64 ± 0.15 0.78 ± 0.09	2.79 ± 0.50 3.49 ± 0.43	2.21 ± 0.34 2.74 ± 0.18	0.35 ± 0.13 0.67 ± 0.05
Fish#4	1.07 ± 0.14 1.50 ± 0.16	0.94 ± 0.30 1.61 ± 0.12	3.88 ± 0.40 3.41 ± 0.15	2.11 ± 0.25 2.84 ± 0.09	0.69 ± 0.26 0.89 ± 0.08
The mean value	1.76 ± 0.10 2.16 ± 0.12	0.65 ± 0.10 1.45 ± 0.14	2.93 ± 0.24 3.01 ± 0.15	2.09 ± 0.14 2.37 ± 0.09	0.54 ± 0.09 0.75 ± 0.04
Freezing time (%)					
Fish#1	20.09 ± 3.20 12.03 ± 1.06	93.63 ± 2.79 41.75 ± 3.70	17.36 ± 12.21 10.92 ± 3.08	28.13 ± 6.39 18.57 ± 3.60	72.90 ± 7.79 73.51 ± 3.70
Fish#2	28.01 ± 5.07 29.53 ± 2.94	81.31 ± 6.15 68.21 ± 3.63	31.55 ± 8.83 12.84 ± 1.53	40.98 ± 8.44 37.03 ± 3.78	90.78 ± 3.01 80.20 ± 2.45
Fish#3	20.61 ± 2.79 13.32 ± 0.96	84.01 ± 4.86 78.24 ± 2.67	11.68 ± 5.83 9.80 ± 6.08	22.92 ± 7.72 8.00 ± 1.51	90.55 ± 4.90 79.24 ± 1.91
Fish#4	59.63 ± 6.03 45.55 ± 4.12	68.49 ± 12.36 54.28 ± 3.89	6.47 ± 3.98 6.89 ± 1.39	23.71 ± 5.95 8.79 ± 1.43	78.33 ± 12.15 71.74 ± 3.70
The mean value	32.08 ± 3.21 25.11 ± 2.36	81.86 ± 3.95 60.62 ± 3.31	16.77 ± 4.34 10.11 ± 1.72	28.94 ± 3.64 18.10 ± 2.18	83.14 ± 3.97 76.17 ± 1.61
Swimming time (%)					
Fish#1	79.35 ± 3.11 85.95 ± 0.97	6.25 ± 2.70 56.61 ± 3.39	81.68 ± 12.02 88.44 ± 3.00	70.03 ± 7.06 81.09 ± 3.57	27.06 ± 7.77 26.45 ± 3.69
Fish#2	71.96 ± 5.07 70.35 ± 2.93	18.41 ± 6.11 31.47 ± 3.65	68.26 ± 8.92 85.89 ± 1.46	58.53 ± 8.30 62.82 ± 3.76	9.17 ± 2.99 19.70 ± 2.41
Fish#3	79.23 ± 2.77 86.45 ± 0.95	15.80 ± 4.82 21.62 ± 2.60	87.93 ± 5.72 87.57 ± 5.67	76.24 ± 7.56 91.45 ± 1.45	9.44 ± 4.90 20.67 ± 1.92
Fish#4	40.06 ± 5.96 54.21 ± 4.14	31.34 ± 12.27 45.49 ± 3.90	91.56 ± 3.80 91.39 ± 1.31	75.97 ± 5.86 90.65 ± 1.40	21.53 ± 12.15 28.13 ± 3.72
The mean value	67.65 ± 3.19 74.24 ± 2.31	17.95 ± 3.92 38.80 ± 3.20	82.36 ± 4.26 88.32 ± 1.62	70.19 ± 3.66 81.50 ± 2.15	16.80 ± 3.96 23.74 ± 1.61
Rapid movement time (%)					
Fish#1	0.56 ± 0.13 2.03 ± 0.30	0.12 ± 0.09 1.64 ± 0.52	0.96 ± 0.24 0.67 ± 0.15	1.85 ± 0.85 0.33 ± 0.05	0.05 ± 0.02 0.04 ± 0.02
Fish#2	0.02 ± 0.02 0.12 ± 0.04	0.29 ± 0.12 0.32 ± 0.06	0.19 ± 0.14 1.27 ± 0.31	0.49 ± 0.18 0.15 ± 0.05	0.05 ± 0.05 0.10 ± 0.04
Fish#3	0.16 ± 0.06 0.22 ± 0.06	0.19 ± 0.12 0.14 ± 0.10	0.39 ± 0.21 2.63 ± 1.27	0.83 ± 0.46 0.54 ± 0.24	0.01 ± 0.01 0.08 ± 0.02
Fish#4	0.31 ± 0.16 0.24 ± 0.11	0.17 ± 0.11 0.23 ± 0.04	1.97 ± 0.75 1.72 ± 0.47	0.32 ± 0.18 0.56 ± 0.14	0.15 ± 0.07 0.13 ± 0.03
The mean value	0.26 ± 0.06 0.65 ± 0.14	0.19 ± 0.05 0.58 ± 0.18	0.88 ± 0.24 1.57 ± 0.36	0.87 ± 0.26 0.40 ± 0.07	0.06 ± 0.02 0.09 ± 0.01
Meandering (°/cm)					
Fish#1	58.29 ± 3.51 45.46 ± 1.66	101.93 ± 15.01 63.16 ± 5.39	31.00 ± 6.04 32.29 ± 1.30	26.09 ± 2.16 37.04 ± 2.03	76.76 ± 14.60 119.31 ± 23.26
Fish#2	31.65 ± 2.23 35.13 ± 2.40	57.87 ± 17.30 76.49 ± 12.58	23.83 ± 2.12 28.85 ± 2.75	43.72 ± 11.87 34.02 ± 2.29	68.34 ± 6.90 56.54 ± 6.03
Fish#3	23.99 ± 2.88 28.77 ± 1.12	61.21 ± 15.98 54.42 ± 5.55	25.31 ± 1.64 43.15 ± 10.36	42.65 ± 10.51 30.77 ± 1.43	80.05 ± 32.96 59.14 ± 17.93
Fish#4	47.67 ± 10.43 51.15 ± 8.07	53.57 ± 8.86 51.59 ± 5.50	18.91 ± 1.10 20.71 ± 0.64	25.72 ± 2.39 29.13 ± 2.68	73.19 ± 10.43 69.23 ± 8.82
The mean value	40.40 ± 3.42 40.13 ± 2.45	68.64 ± 7.94 61.42 ± 4.27	24.76 ± 1.80 31.25 ± 3.03	34.55 ± 4.11 32.74 ± 1.13	74.59 ± 8.94 76.05 ± 9.01
Wall preference index					
Fish#1	0.79 ± 0.07 0.81 ± 0.02	0.08 ± 0.06 0.57 ± 0.05	0.62 ± 0.09 0.49 ± 0.03	0.44 ± 0.07 0.53 ± 0.04	0.57 ± 0.16 0.61 ± 0.08
Fish#2	0.41 ± 0.05 0.52 ± 0.03	0.18 ± 0.10 0.29 ± 0.04	0.44 ± 0.04 0.51 ± 0.03	0.41 ± 0.09 0.34 ± 0.04	0.09 ± 0.07 0.26 ± 0.07
Fish#3	0.48 ± 0.04 0.59 ± 0.04	0.34 ± 0.10 0.30 ± 0.04	0.40 ± 0.13 0.59 ± 0.09	0.39 ± 0.11 0.51 ± 0.06	0.49 ± 0.15 0.57 ± 0.09
Fish#4	0.45 ± 0.09 0.49 ± 0.04	0.29 ± 0.15 0.45 ± 0.04	0.64 ± 0.08 0.57 ± 0.04	0.45 ± 0.08 0.63 ± 0.03	0.62 ± 0.15 0.36 ± 0.06
The mean value	0.53 ± 0.04 0.60 ± 0.02	0.22 ± 0.05 0.40 ± 0.03	0.52 ± 0.05 0.54 ± 0.03	0.42 ± 0.04 0.50 ± 0.03	0.44 ± 0.08 0.45 ± 0.05

Note: characteristics are given as mean ± mean error; FDGV—fluctuations of diurnal geomagnetic variation.

**Table 2:** Parameters of locomotor activity in zebrafish under geomagnetic disturbances (above dash) and at the same time on magnetically quiet days (below dash) in summarized groups.

Parameter	Events after FDGV n = 8	Event before FDGV n = 8	Event Coincided with FDGV n = 4
Avg. swimming speed (cm/s)	1.93 ± 0.17 2.27 ± 0.24 *	0.60 ± 0.08 1.10 ± 0.20 †	2.93 ± 0.35 3.01 ± 0.26
Freezing time (%)	30.51 ± 4.78 21.60 ± 4.99 **	82.50 ± 3.18 68.40 ± 4.82 *	16.77 ± 5.41 10.11 ± 1.24
Swimming time (%)	68.92 ± 4.76 77.87 ± 4.89 **	17.37 ± 3.17 31.27 ± 4.66 *	82.36 ± 5.12 88.32 ± 1.15
Rapid movement time (%)	0.57 ± 0.20 0.53 ± 0.22	0.13 ± 0.03 0.34 ± 0.19	0.88 ± 0.40 1.57 ± 0.42
Meandering (°/cm)	37.47 ± 4.41 36.43 ± 2.84	71.62 ± 5.43 68.74 ± 7.78	24.76 ± 2.49 31.25 ± 4.65
Wall preference index	0.48 ± 0.05 0.55 ± 0.05 *	0.33 ± 0.07 0.43 ± 0.05	0.52 ± 0.06 0.54 ± 0.02

Note: characteristics are given as mean ± mean error; FDGV—fluctuations of diurnal geomagnetic variation; n—sample size; significant differences compared with the control (paired-samples *t*-test): ** *p* < 0.01, * *p* < 0.05. † Marginal differences *p* < 0.1.

## Data Availability

Data are available as Supplementary Files.

## Supplementary Materials

The following are available online at https://www.mdpi.com/article/10.3390/clockssleep3040045/s1.
