# Supplementary material for: Influence of Geomagnetic Disturbances at Different Times of Day on Locomotor Activity in Zebrafish (Danio Rerio)
Source: Clocks Sleep. 2021 Nov 29;3(4):624–32. doi: 10.3390/clockssleep3040045 (PMC8700499; doi:10.3390/clockssleep3040045)
Supplement: Supplementary file 1 [file clockssleep-03-00045-s001.zip › Supplementary Figure S1.pdf]

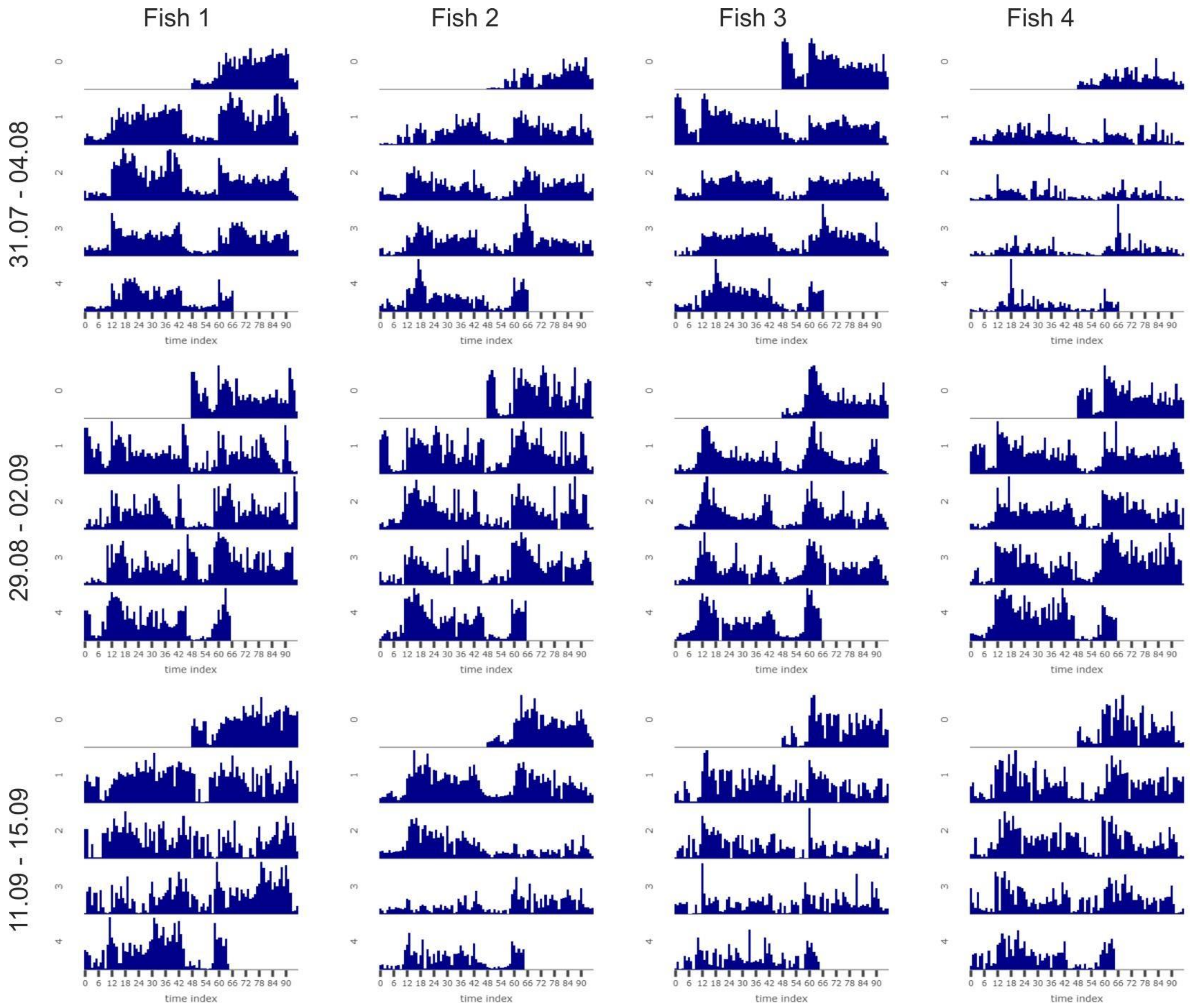


**Supplementary Figure S1.** Unsmoothed double plotted actograms of each zebrafish locomotor activity (swimming speed).
